# The Long-Term Care Insurance Program in Israel: solidarity with the elderly in a changing society

**DOI:** 10.1186/2045-4015-2-3

**Published:** 2013-01-23

**Authors:** Sharon Asiskovitch

**Affiliations:** 1 Social Policy Analyst, Research and Planning Administration, Israel National Insurance Institute, 13 Weizmann Blvd 91909, Jerusalem, Israel

**Keywords:** Long-term care, Long-term care insurance program, Home-based personal care, Israel, Solidarity, Beneficiaries, Benefits

## Abstract

The Long-Term Care Insurance Program (LTCIP) in Israel is a social security program administered by the National Insurance Institute (NII) since 1988. LTCIP focuses on home-based personal care services. Differently from most other programs under the responsibility of the NII, LTCIP benefits are in-kind benefits and are delivered via multiple for-profit and not-for-profit organizations. In recent years LTCIP has been the target of various legal amendments and numerous administrative changes. While many of these changes may have had significant effects on individuals, they have not altered the fundamental principles of the program. Thus, many of the characteristics of beneficiaries have remained quite stable over the years; other characteristics of the population of beneficiaries have changed over the years reflecting the aging of Israeli society. A central issue related to LTCIP is whether benefits are adequate to meet the needs of the growing elderly population of Israel. While the generosity of LTCIP benefits is questionable, economic and political struggles have limited the scope of changes introduced thus far.

## Introduction

Home care for the elderly is the main long-term care (LTC) service to the elderly in Israel. In 2011, a monthly average of 145,600 elderly people – constituting about 80% of the elderly in Israel receiving formal assistance – lived in the community. Expenditure on Long-Term Care Insurance Program (LTCIP) benefits in Israel reached NIS 4 billion (slightly over 1 billion US$) in 2011, or 0.5% of Israel’s GDP [1]. Spending for LTCIP represents the majority of public spending on home care and institutional LTC services in Israel – 69% of total public spending in 2010. Spending on LTCIP represented 63% of total spending on LTC services in the community in 2010 [2]: 91.

In the past 15 years, the share of elderly people – age 65 or over – has remained 9.8%-9.9%, after a gradual increase from less than 5% in the 1950s [3]: 18. According to projections of the Israel Central Bureau of Statistics [4]: 53, the share of the elderly population is expected to increase and reach over 14% within the next two decades. Therefore, there is a need to review policies towards the elderly and their implementation. Similarly to the case in other welfare states, the number of elderly people in Israel in need of LTC services is expected to rise, as are the costs of these services. Thus, Israeli society should consider the place and role of LTCIP within the broader framework of social policies and their social and economic outcomes.

Programs like LTCIP have several roles. First, they represent a commitment of society in general to support the non-medical needs of the elderly. Second, public provision of formal care giving based on need serves the goal of enhancing solidarity with the elderly and their families and promoting redistributive justice within a given society. Third, LTCIP is aimed at sharing with family members the burden of care while not entirely replacing the family. In-kind and/or cash benefits assist informal caregivers in coping with the physical, emotional and economic burdens of care giving while strengthening the commitment of families to those in need. Fourth, LTCIP encourages elderly people to keep living in their communities for as long as they can and wish to. Last, LTCIP influences unskilled sections of the labor market as formal caregivers, and due to the massive role of non-citizen labor migrants, it also contests the boundaries of societies and their character.

Social solidarity is the symbolic as well as materialistic willingness of various groups or individuals in society to contribute to the welfare of other groups or individuals, based on a common belief that mutual support/cooperation is needed for the cohesion of society [5]. Decisions over the scope of solidarity – what is included and who is included – result from politics and past decisions [6]. For social programs it means that members of society both make contributions and enjoy rights linked to their needs. As institutions shape social interactions by creating roles and expectations of actors [7], social programs develop bonds among people that include moral commitments and economic exchanges [8]. LTCIP is an institution, and as such, is a mechanism used to organize the distribution of material and/or symbolic values in a realm of life in a given society [7].

LTCIP, at large, has proven to be resilient to radical changes. Since its full introduction in 1988, few major legal reforms have been introduced. Its goals, rules of entitlement, basket of services and relationships among the main actors have not been altered to any great extent. As presented below, the characteristics of the population of beneficiaries have changed over the years to reflect the aging of Israeli society, the aging of the elderly themselves and the growing share of dependent elderly among the aged.

There has been extensive research on LTCIP. Numerous studies have reviewed various aspects of it, including its challenges and achievements [2,9-32])^a^. A small number of comparative studies of the Israeli case were published [33-38]. Attention was also given to the question of whether or not the benefits are generous and adequate [10,11,16-18,22,39,40]^b^.

This article presents several issues regarding beneficiaries and discusses the issue of the generosity of benefits. The first section of the paper briefly outlines the structure of LTCIP as well as its sources of financing and scope of expenditure. The second section presents data regarding trends among the beneficiaries. The third section discusses the issue of the generosity of the program towards its beneficiaries and its implications for the solidarity of Israeli society with its aged population.

The first two sections of the article are a descriptive analysis of the program, and the trends in the data presented will be evaluated to confirm certain elements of the program experience. The third section focuses on evaluating to what extent LTCIP has been able to achieve its main goals of supporting the LTC needs of frail elderly and to ease the burden on family support in the changing social, economic, and cultural environment of Israeli society ^c^.

The topics centering on beneficiaries and distribution of resources discussed in this paper are among the more debated issues in recent years and they do not cover all aspects of LTCIP (see Appendix B). In the last section of the article, I propose some topics for future research, some of which are linked to issues discussed in this article.

## LTCIP: an overview

LTCIP is administered by the National Insurance Institute (NII) ^d^. It covers home-based personal care ^e^. The program is financed through employment-based payroll contributions of both employees and employers, and insurance is mandatory. A share of the cost is financed out of the general revenue ^f^. LTCIP is income-tested with the aim of excluding the highest income earners. Israel’s system of LTC services is an income/means-tested model [35]: 226–229, and one should distinguish between the rules covering home-based care and those applying to formal institutional care, and recognize the exclusionary purpose of income-tests in LTCIP ^g^.

LTCIP came into force in April 1988, 7.5 years after the Knesset, the Israeli Parliament, approved an amendment to the National Insurance Act [NIA]. LTCIP, a program that has increased spending for LTC services for the elderly in the long run, was legislated at a time of economic austerity. At that time, from an economic point of view, it was meant to weaken the pressures for larger funding for institutional care with the promise to develop a much cheaper framework for community care [18].

LTCIP is based on several principles. It is a social security program^h^. The program grants frail elderly people in-kind benefits via not-for-profit and for-profit providers contracting with the NII within a quasi-market setting. Less than 1% of beneficiaries receive cash benefits, most of them as part of an experimental program (see below) [41]. Services include assistance at home and some household management (such as cleaning and cooking) [42], care in day centers for the elderly, absorbent undergarments, personal alarm units and laundry services. Almost all beneficiaries receive assistance at home and for many this is the sole service financed by the NII [1]: 135.

Several rules of entitlement govern eligibility: the claimant must be an Israeli citizen or a permanent resident; the claimant must be above the retirement age, i.e. 67 for men and 62 for women; the claimant must live at home; the claimant must not receive an equivalent state-funded benefit; the claimant and spouse must pass an income test (savings, real estate, and property are not included) ^i^; the claimant must pass a dependency test. The type of services and the identity of the providers are determined by a local LTC committee composed of a nurse, a social worker on behalf of the municipality and a NII claim officer [30]: 237. The beneficiary (or her family) can ask for a certain service or provider and the local LTC committee accepts these wishes in most cases.

The total score in the dependency test is composed of three parts (see Table 1) ^j^. The first considers the extent to which one requires the help of others ^k^ to perform basic activities of daily living (ADL) ^l^: bathing; dressing; eating (and cooking) ^m^; mobility in the home and the occurrence of falls; control of urine and bowel movements ^n^. The second part considers the need for permanent or partial supervision due to cognitive, psychological or physical limitations ^o^. The third part is an additional score for people living alone, as it is assumed that they require greater formal care giving. The total dependency score is the highest of the scores in the first two parts plus the additional score for living alone. Thus, the dependency score can run between 0 and 11.

**Table 1:** Components of the dependency test

**Criteria for assessment**	**Score / Range of scores**
ADL	0-8.5 in leaps of 0.5 (9 in rare cases can an individual score 1 in both ‘falls’ and ‘mobility’)
Occurrence of falls	0-1
Mobility in the home	0-1
Dressing	0-1
Bathing	0-1.5
Eating and cooking	0-1.5
Control of urine and bowel movements	0-1, 2-3
Need for supervision	0, 4 (partial; 2.5 until 31/12/2011), 9 (constant)
Living alone	0.5 (for those with 0–4 points in other parts), 1 (for 85 years-old blind persons living alone), 2 (for those with 4.5-9 points in other parts)
Total	0-11

Source: [1]: 125; [43].

Table 2 presents the distribution of ADL limitations and their severity among beneficiaries in December 2011 as evaluated by their dependency score. The distribution of limitations seem to coincide with the gradual process of ADL deterioration.

**Table 2:** Distribution of LTCIP beneficiaries by score in various activities of daily living and need of supervision, December 2011 (in %)

**Score / Activity**	**Bathing**	**Dressing**	**Mobility in the home**	**Occurrences of falls**	**Control of urine and bowel movements**	**Eating and cooking**	**Need of Supervision**
0	0.4	1.0	54.7	78.5	29.6	5.7	80.4
0.5	27.3	8.8	33.3	17.6	18.4	82.6	-
1	47.3	90.2	12.0	3.9	16.4	8.0	-
1.5	25.0	-	-	-	-	3.7	-
2	-	-	-	-	14.4	-	-
2.5	-	-	-	-	11.5	-	2.5
3	-	-	-	-	9.7	-	-
9	-	-	-	-	-	-	17.1
Total	100.0	100.0	100.0	100.0	100.0	100.0	100.0

Source: [1]: 126.

Note: Data do not include beneficiaries who died or moved to an institution during December 2011 or beneficiaries whose eligibility was determined in a “fast track” procedure (see below).

Table 3 presents the average score in each activity of daily living for each dependency score among beneficiaries in December 2011 (the need for supervision and an additional score for people who live alone are included). One can see the process by which one’s disabilities and their severity widen as one’s dependency in old age increases. As for most of those with 9 points and for almost all those with 11 points – their dependency is determined by their need of supervision. Thus, their range of physical disabilities is quite wide.

**Table 3:** Average scoring in activities of daily living by dependency score

**Dependency score**	**Number of individuals**	**Bathing**	**Dressing**	**Mobility in the home**	**Occurrences of falls**	**Control of urine and bowel movements**	**Eating and cooking**	**Need of Supervision**	**Living Alone**
2.5	34,478	0.70	0.91	0.01	0.06	0.15	0.42	0.06	0.25
3	21,962	0.81	0.94	0.03	0.07	0.29	0.47	0.15	0.33
3.5	10,088	0.84	0.97	0.13	0.12	0.65	0.50	0.07	0.28
4	6,469	0.93	0.98	0.31	0.12	0.92	0.51	0.07	0.23
4.5	4,259	0.98	0.98	0.40	0.17	1.13	0.52	0.06	0.31
5	2,209	0.99	0.99	0.50	0.08	1.91	0.52	0.06	0.00
5.5	1,098	1.15	0.99	0.55	0.14	2.14	0.54	0.07	0.07
6	5,267	1.31	1.00	0.62	0.18	2.34	0.56	0.06	0.00
6.5	10,065	1.20	0.99	0.52	0.26	1.78	0.58	0.06	1.16
7	8,852	1.13	0.99	0.57	0.14	2.12	0.58	0.05	1.46
7.5	5,482	1.20	1.00	0.59	0.19	2.24	0.62	0.06	1.66
8	4,239	1.31	1.00	0.64	0.22	2.43	0.67	0.07	1.74
8.5	2,399	1.39	1.00	0.72	0.23	2.56	0.63	0.05	1.96
9	15,282	1.24	0.92	0.48	0.15	1.84	0.73	7.38	0.36
9.5	1,056	1.49	1.00	0.89	0.28	2.80	1.03	0.08	2.00
10	600	1.50	1.00	0.96	0.22	2.95	1.37	0.09	2.00
10.5	60	1.49	1.00	0.93	0.76	2.93	1.39	0.13	2.00
11	12,436	1.13	0.87	0.33	0.15	1.42	0.68	8.99	2.00

Source: [1]: 127.

Note: Data do not include beneficiaries who died or moved to an institution during December 2011 or beneficiaries whose eligibility was determined in a “fast track” procedure (see below).

The income test makes use of two thresholds. Individuals with no spouse are entitled to a full benefit if their monthly income does not exceed the average wage and to half the benefit if their monthly income does not exceed 1.5 times the average wage. Individuals with spouses are entitled to a full benefit if their and their spouse’s monthly income does not exceed 1.5 times the average wage, and to half the benefit if their and their spouse’s monthly income does not exceed 2.25 times the average wage.

Since January 2007 three levels of benefits exist. A total score of 2.5 points in the dependency test is required to qualify for a benefit. Individuals living alone require 2 points in their assessment to qualify as they receive an additional 0.5 point (see Table 1). Levels of benefits are calculated as percentages of a full general disability pension for a person without dependents (about a quarter of the average wage). The levels of benefits are translated into weekly home care hours: Individuals with a total score of 2.5–5.5 points, 6–8.5 points and 9 points or more in the dependency test receive a benefit at levels of 91%, 150% and 168%, respectively, equivalent to 9.75, 16 and 18 weekly hours, respectively. Individuals entitled to half the benefit due to income-testing are entitled to half the number of weekly hours. As an incentive for employing Israeli caregivers, beneficiaries who are entitled to benefit at the 150% or 168% levels receive additional 3 and 4 weekly home care hours, respectively. The distribution of beneficiaries in terms of the number of weekly home care hours they received as of December 2011 is shown in Table 4.

**Table 4:** Distribution of permits for employing foreign workers as caregivers by LTC benefit level, December 2011 compared to December 2010

**Benefit level**	**Number of weekly hours of home care provided**	**December 2010**			**December 2011**		
**(N)**		**Have a valid permit**	**Do not have a valid permit**	**Total**	**Have a valid permit**	**Do not have a valid permit**	**Total**
45.5%	5	710	3,340	4,050	638	3,566	4,204
91%	9.75	2,672	72,658	75,330	2,243	74,664	76,907
75%	8/9.5	1,598	745	2,343	1,563	895	2,458
150%	16/19	15,000	18,584	33,584	14,745	20,141	34,886
84%	9/11	1,542	582	2,124	1,584	669	2,253
168%	18/22	15,147	11,218	26,365	15,807	12,512	28,319
Total		36,669	107,127	143,796	36,580	112,447	149,027
**(% of total among benefit level group)**							
45.5%	5	17.5	82.5	100.0	15.2	84.8	100.0
91%	9.75	3.5	96.5	100.0	2.9	97.1	100.0
75%	8/9.5	68.2	31.8	100.0	63.6	36.4	100.0
150%	16/19	44.7	55.3	100.0	42.3	57.7	100.0
84%	9/11	72.6	27.4	100.0	70.3	29.7	100.0
168%	18/22	57.5	42.5	100.0	55.8	44.2	100.0
Total		25.5	74.5	100.0	24.5	75.5	100.0

Source: NII – Research and Planning Administration.

At the end of 2011 the beneficiaries in 9 regions (i.e. local branches of the NII) out of a total of 23 regions were allowed to choose between in-kind and cash benefits if they meet following criteria [44]: they live in one of the 9 pilot regions, they are entitled to benefit at the 150% or 168% benefit level (or half those levels), they employ formal caregivers for no fewer than 6 days a week, 12 hours a day, and they present a signed contract with a formal caregiver who is not a family member. If the formal caregiver is a non-Israeli labor migrant, the claimant must possess a valid permit from the Population and Immigration Bureau of the Ministry of the Interior (MOI). People receiving cash benefits receive 80% of the equivalent in-kind benefits. The gap in values represents the service providers’ costs of operating their services and the additional taxes that they are obliged to pay. Studies by the NII found out that beneficiaries who receive cash benefits enjoyed a similarly high quality of care as did beneficiaries who receive services from LTC agencies [19]: 16–18; [45].

In recent years various amendments to LTCIP were introduced. Some amendments considered radical at first glance are seen to be more moderate when considered over time ^p^: the introduction of a third, higher, level of benefit in 2007 ^q^ has had a minor impact since the gap between the two highest benefit levels is rather small; the introduction of cash benefits since 2008 ^r^ has been enjoyed by a relatively small share of beneficiaries; increasing benefits to beneficiaries employing formal Israeli caregivers since 2009 ^s^ has had a minor impact due to the fact that the real cost of employing a non-Israeli caregiver has not changed as well as due to other factors that have affected the incentive for employing non-Israeli formal caregivers (see Appendix B).

Other legal changes are related to rules of eligibility. The retirement age for men and women was gradually increased during 2004–2008 – from 60 to 62 for women and from 65 to 67 for men. However, this too is not a radical change. The under-70 age group has consistently included only 1% or fewer LTCIP beneficiaries. Moreover, disabled individuals under the retirement age may apply for an attendance allowance, which for some may prove to be more generous than an equivalent LTCIP benefit ^t^. Among other changes in the rules of eligibility are granting all 90 years-old or over the option to be evaluated by a physician instead of a nurse, and later expanding it to 80–89 years-old in three regions as a pilot plan, change in income tests to exclude some financial sources of Holocaust survivors, extending the duration of the experimental program for cash benefits, and granting entitlements to beneficiaries temporarily hospitalized ^u^. Many of these changes affecting specific groups of claimants or beneficiaries were proposed by politicians, since a public outcry against how some elements of this program, such as the dependency tests (see below), have been administered, generates increased involvement of politicians. LTCIP has been a considered by some politicians to be a favorable realm to promote the welfare of the elderly population. LTCIP is a popular social program, as younger people are familiar with experiences of family members and/or realize that future dependency at old age is common to all.

Various components of LTCIP are determined by procedures articulated by the NII as the law is applied into real-life. Two examples are the dependency test and the dependency scale. The content of the dependency test is based on comparative experience in other countries and has been examined from time to time by committees that included experts in geriatric medicine [46,47]. The exact procedures of the test are set by the professional department at the NII [43]. Furthermore, the dependency scale is not included in the NIA. The law asserts that dependency should be based on severe physical limitations or on a need for supervision, and sets three levels of benefits (or two prior to 2007), labeling them with vague titles. It has been the responsibility of the NII to decide how dependency is measured and scaled, to set a lower threshold for eligibility, to determine how some situations such as living alone should be evaluated, and so forth.

Some changes in the structure and content of the dependency test in recent years have been due to complaints of claimants against practices they considered to be degrading and offensive. In the dependency test, claimants may be asked to demonstrate several activities of daily living. Some parts of the dependency test might be considered by some claimants as intrusive or even degrading. Dressing and bathing seem to be the most difficult parts of the dependency test from the claimants’ point of view [48]. Following the Brill Committee in 2005 [47], in recent years, nurses have been asked to base their evaluations on “indirect observations” of claimants’ behavior rather than on claimants’ demonstrations of activities of daily living, whenever possible [43]. It should be emphasized that the nurses conducting the dependency test are instructed not to ask claimants to undress, and demonstrations include wearing clothes over clothes and washing one’s hands in order to ensure claimants’ dignity [43]. Similarly, evaluation of “control of urine and bowel movements” is based on other sections of the dependency test such as mobility in the home and dressing, as well as questions presented to the claimants and/or relatives [43].

In 2005 a survey conducted on behalf of a public committee set to examine the dependency test in LTCIP [47] found out that among 294 claimants, satisfaction with various parts of the functional evaluation and the attitudes of the nurses performing the evaluations was correlated with the outcome of one’s claim. While 83.3%-89.3% of respondents whose claim was accepted answered that they felt comfortable with different parts of the evaluation, only 63.1%-71.5% of the respondents whose claim was rejected felt the same. As for the respondents’ evaluations of the nurses performing the evaluations, 84.9%-97.8% of the respondents whose claim was accepted argued that the nurses treated them with respect, while only 70.4%-91.5% of the respondents whose claim was rejected felt the same. The rates of approval of various parts of the dependency test among those who did not receive a decision prior to the survey were closer to those whose claim was approved [48].

Other changes in the dependency evaluation process include the introduction of a “fast track” procedure replacing the dependency test: based on medical documentation, people with very severe disabilities such as advanced dementia receive the highest benefit [49]. Also, a decision to increase the score in the dependency test for a group of 85 years-old – or over – lonely blind people resulted from a prior decision of the NII in one case that led to public criticism of the NII [50]. In 2005, due to a ruling of the National Labor Court, the NII changed the definition of ‘living alone’ in the dependency test to include persons who live with others who cannot take care of themselves (such as spouses eligible for LTCIP or disabled children) [51].

### Expenditure

LTCIP has become one of the fastest growing programs administered by the NII in terms of expenditure. In just over two decades the share of LTCIP out of NII’s total expenditure on social security and income support increased from 2% to 6.9% (see Table 5). From 1989 to 2011 expenditure on LTCIP increased at about 8.2 times – from NIS 511 million to NIS 4,213 million (in 2011 prices) compared to an increase in the monthly average number of recipients – about 6.8 times ^v^.

**Table 5:** Expenditure on LTCIP (various years; 2011 prices)

**Year**	**NII total expenditure on benefits [I] (NIS million)**	**NII total expenditure on LTCIP [II]**	**II as % of I**	**Net expenditure on LTCIP benefits (NIS million) [III]**	**III as % of II**	**Average monthly benefit recipients (000s)**
1989	25,778	511	2.0	384	75.2	21.4
1992	28,562	858	3.0	715	83.3	37.7
1996	38,395	1,376	3.6	1,187	86.3	66.0
1999	45,989	1,930	4.2	1,721	89.1	88.2
2001	56,384	2,708	4.8	2,464	91.0	104.2
2002	55,408	2,995	5.4	2,769	92.5	112.3
2003	52,167	2,926	5.6	2,709	92.6	113.0
2006	51,927	3,137	6.0	2,927	93.3	120.4
2007	52,881	3,527	6.7	3,307	93.7	125.4
2008	53,607	3,622	6.8	3,425	94.5	131.1
2009	57,643	3,909	6.8	3,628	92.8	136.4
2010	59,942	4,134	6.9	3,908	94.5	141.3
2011	61,317	4,213	6.9	3,996	94.9	145.6

Source: [1]: 122, 135; [41].

Over the years, the share of insurance contributions and Ministry of Finance (MOF) contributions ^w^ has decreased. Thus, the NII has to allocate, according to the NIA, funds from other programs to finance the rising costs of LTCIP. The sum of insurance fees and government’s contributions out of total sources of financing fell from 43% in 1994 to 34.7% in 2011. During 1994–2011 the share of insurance fees in financing LTCIP gradually fell from 27.8% in 1994 to 14.9% in 2002. In 2011 the share of contributions fees was 14.1%. The share of MOF contributions increased from 15.3% in 1994 to 23.3% in 2002 due to the wave of immigration from the FIS. Since 2004 the share of MOF contributions fell (except in 2009) to 20.6% of total financing in 2011, as the share of uninsured immigrant elderly in the Israeli society gradually decreased. The share of NII in financing LTCIP (transfers of surpluses from other branches of NII, mainly from the Children branch, to LTCIP) has increased from 57% in 1994 to 65.3% in 2011.

## Trends in LTCIP: beneficiaries

There are several groups that benefit from the resources of LTCIP. The most obvious group is that of the frail elderly in need of LTC services. Other groups are service providers and their employees, including formal caregivers, who are discussed in Appendix B.

The monthly average number of LTCIP beneficiaries increased by 6.6 times in just two decades – from 21,359 in 1989 to 141,382 in 2010. Table 6 presents the trends of growth in the number of beneficiaries and the number of people aged over retirement age. During this period, the relative number of LTCIP beneficiaries increased faster than did the total number of elderly people. Thus, the take-up rate among the elderly population has steadily increased. The take-up rates since 2004 are actually higher than the figures presented in Table 6^x^. Thus, in 2010 the real take-up rate was 17.4%.

**Table 6:** Elderly people and LTCIP beneficiaries, 1995–2010 (in 000s)

**Year**	**Monthly average elderly people**	**Change since last year**	**Change in %**	**Monthly average LTCIP beneficiaries**	**Change since last year**	**Change in %**	**Take-up rate (%)**
1995	624.0	-	-	59.0	-	-	9.5
1996	641.4	17.4	2.8	66.0	7.0	11.9	10.3
1997	679.3	37.9	5.9	72.9	6.9	10.5	10.7
1998	695.7	16.4	2.4	81.0	8.1	11.1	11.6
1999	711.5	15.8	2.3	88.2	7.2	8.9	12.4
2000	728.7	17.2	2.4	95.8	7.6	8.6	13.1
2001	744.5	15.8	2.2	105.4	9.6	10.0	14.2
2002	758.1	13.6	1.8	112.3	6.9	6.5	14.8
2003	769.4	11.3	1.5	113.0	0.7	0.6	14.7
2004	780.6	11.2	1.5	113.4	0.4	0.4	14.5
2005	794.8	14.2	1.8	115.0	1.6	1.4	14.5
2006	813.9	19.1	2.4	120.5	5.5	4.8	14.8
2007	836.4	22.5	2.8	125.4	4.9	4.1	15.0
2008	859.1	22.7	2.7	131.1	5.7	4.5	15.3
2009	895.7	36.6	4.3	136.4	5.3	4.0	15.2
2010	925.2	29.5	3.3	141.4	5.1	3.7	15.3

Source: [41,52].

Note: Take-up rates among women aged 60 and over and men aged 65 and over.

Various factors can explain the growth in the number of LTCIP beneficiaries. The growth of general elderly population is one factor. Table 6 reveals a similarity between annual changes in the total number of elderly people in Israel and the total number of LTCIP beneficiaries during 1996–2010. 1997 and 2009 seem to be exceptions. The gap has increased since 2004 as the retirement ages for women and men were steadily increased.

Within the general elderly population, the aging of the elderly population itself is a factor influencing the size and scope of dependency, as levels of dependency increase with age. Trends of age and dependency levels among beneficiaries are discussed below. Also, the steadily rising take-up rates can be ascribed to the widening awareness among the elderly population, as well as among younger family members, of their rights to LTCIP, the efforts of associations for the rights of the aged and LTC agencies to spread information about the program, and the intensified public debate about LTCIP’s rules of eligibility and generosity of benefits, as well as to the efforts of the NII to inform the public about the program.

Trends in the composition of age and gender of the elderly population in Israel are mirrored to some extent by LTCIP beneficiaries [53]. As for age groups, the share of people aged 85 or over out of the total elderly population increased from 9% in 2000 to 10.8% in 2010. In the same period, the share of LTCIP beneficiaries aged 85 or over increased from 33.2% to 36.9%. As for gender groups, the share of women among the elderly population slightly increased: from 63.6% in 1995 to 64.8% in 2010. At the same period, the share of women among LTCIP beneficiaries has slightly decreased: from 71.8% to 70.9%.

Female and male beneficiaries have, on average, different characteristics, which may shed some light on their specific needs of LTC assistance and services. Among women aged 62 years-old or over, 18.7% receive LTCIP benefits, while only 14.5% of men aged 67 years-old or over receive LTCIP benefits. Table 7 presents the distribution of LTCIP beneficiaries in December 2011 according to gender, benefit level and living with or with no spouse. Most female beneficiaries live with no spouse, while most male beneficiaries live with a spouse. This difference between women and men can be explained by the life expectancy gap between them, as women live longer ^y^.

**Table 7:** Distribution of LTCIP beneficiaries according to gender, benefit level and living with or with no spouse, December 2011 (in %)

**Benefit level**	**45.5%**	**75%**	**84%**	**91%**	**150%**	**168%**	**Total**
**Female**							
With no spouse	60.2	71.5	63.1	67.6	74.0	72.2	69.8
With a spouse	39.8	28.5	36.9	32.4	26.0	27.8	30.2
Total	100.0	100.0	100.0	100.0	100.0	100.0	100.0
N	2,625	1,429	1,315	55,513	25,034	19,885	105,801
**Male**							
With no spouse	40.3	42.3	36.5	36.0	36.4	33.7	36.0
With a spouse	59.7	57.7	63.5	64.0	63.6	66.3	64.0
Total	100.0	100.0	100.0	100.0	100.0	100.0	100.0
N	1,579	1,029	938	21,394	9,851	8,434	43,225

Source: NII – Research and Planning Administration.

The average age of LTCIP beneficiaries in December 2011 was 82.1. The average age of female beneficiaries was 81.7, and that of male beneficiaries – 82.8. The average age of beneficiaries living with a spouse was 80.6, and that of beneficiaries living with no spouse was 83.0. The average ages of beneficiaries receiving benefit at the lowest, medium and highest benefit levels were 80.6, 83.7, and 83.9, respectively. The age gap between the lowest level and the two higher levels represents the relationship between age and greater need for long-term care. Table 8 presents the average age of each of the groups identified in Table 7. While most men, unlike women, live with a spouse their need of formal care is greater as they are older and their spouses (as well as other relatives) cannot supply all needed care giving. Female beneficiaries tend to receive benefits for a longer time than do men (51.3 months compared to 46.1 months), as men tend to enter the system, on average, at a later point of their lives (78.8 for men and 75.9 for women) ^z^.

**Table 8:** The average age of LTCIP beneficiaries according to gender, benefit level and living with or with no spouse, December 2011

**Benefit level**	**45.5%**	**75%**	**84%**	**91%**	**150%**	**168%**	**Total**
**Female (all beneficiaries; N=105,801)**							
With no spouse	82.0	85.3	84.6	81.1	84.6	84.9	82.8
With a spouse	77.1	78.9	79.8	78.0	80.9	81.3	79.2
Gap	4.9	6.4	4.8	3.1	3.7	3.6	3.6
**Female (aged 67 or over; N=102,896)**							
With no spouse	82.6	85.6	84.7	81.6	84.8	85.1	83.2
With a spouse	78.6	79.9	80.5	78.8	81.3	81.7	79.9
Gap	4.0	5.7	4.2	2.8	3.5	3.4	3.3
**Male (all beneficiaries; N=43,225)**							
With no spouse	83.9	85.7	85.1	82.5	85.4	85.5	83.9
With a spouse	81.2	82.4	82.5	81.5	83.2	83.2	82.2
Gap	2.7	3.3	2.6	1.0	2.2	2.3	1.7

Source: NII – Research and Planning Administration.

The aging of LTCIP beneficiaries translates into changes in the composition of benefit level groups as the incidence and scope of limitations in ADL and the need for supervision, due, for example, to dementia, increase. In 1990, 24.3% of beneficiaries were eligible for the higher benefit. Since 1998 the share of the beneficiaries eligible for benefit at the higher level – or since 2007 at the two highest levels – increased from 21.8% to 45.1% in 2011. Many of the recipients of one of the two highest levels are individuals whose LTC needs increased over the years and their benefits were raised thereafter. 36.9% of beneficiaries entered LTCIP in December 2011 with a lower benefit. This is expected, since the average duration of eligibility for LTCIP benefits was 49.4 months in December 2011, based on NII data ^a.a^.

## LTCIP: generosity and solidarity

A major concern regarding LTCIP has been the adequacy of its benefits, especially for those in need of assistance and/or supervision for most hours of the day. Are the resources of LTCIP allocated adequately: do the severely frail receive enough home care? Answers may be given from different perspectives. One is from within the program – whether people experiencing different levels of dependencies enjoy various benefits adequate to their condition of dependency. Another way to evaluate LTCIP is by comparing it to programs in other welfare regimes.

The main goal of LTCIP has been to ease the physical and emotional, as well as financial, burden of caring for older family members, not to substitute for the family as a prime source of care giving. However, as both the number of beneficiaries and expenditure levels have increased over the years and the costs of care giving for families have risen as well, the question of whether the structure of benefits should be amended has become a pressing concern for decision-makers [31].

The LTCIP benefit system is based on a weak positive correlation between dependency score and benefit level [1]: 129; [30]: 235–236; [54]. First, severely dependent persons are eligible for fewer weekly home care hours per additional point of dependency; those with 2.5 points enjoy 3.9 weekly hours per point while those with 11 points enjoy 2 or 1.64 weekly hours per point. Second, the benefit system is quite regressive. Furthermore, one can compare the benefits of two individuals: one with 2.5 points and another with 5.5 points: both are entitled to the same amount of weekly home care hours – 9.75 – although one certainly needs greater assistance than the other. Third, the current system is nonlinear, as exemplified by the jump between 5.5 and 6 points. Such nonlinearity might act as an incentive to “push” individuals located in these “borderlines situations” onto higher levels on the scale to increase their benefits, or lower levels on the scale to rollback expenditures.

Until recent years, the dependency score represented the “desired” number of daily hours of home care needed to help a frail elderly individual with activities of daily living or with supervision; each 0.5 point represented a daily half an hour required for helping one’s activity of daily living, based on analysis of experts [39,54]. Table 9 shows the gaps between the granted levels of benefits and the “desired” levels (weekly hours = daily hours * 7), as perceived in the past.

**Table 9:** Granted and “desired” weekly home care hours

**Dependency score**	**Granted hours**[1]	**“Desired hours”**[2]	[1]**as % of**[2]
2.5	9.75	17.5	55.7
3	9.75	21	46.4
3.5	9.75	24.5	39.8
4	9.75	28	34.8
4.5	9.75	31.5	31.0
5	9.75	35	27.9
5.5	9.75	38.5`	25.3
6	16/19	42	38.1/45.2
6.5	16/19	45.5	35.2/41.8
7	16/19	49	32.7/38.8
7.5	16/19	52.5	30.5/36.2
8	16/19	56	28.6/33.9
8.5	16/19	59.5	26.9/31.9
9	18/22	63	28.6/34.9
9.5	18/22	66.5	27.1/33.1
10	18/22	70	25.7/31.4
10.5	18/22	73.5	24.5/29.9
11	18/22	77	23.4/28.6

In recent years, the dependency score merely represents the level of dependency of a person in various activities of daily living based on a structured dependency evaluation [43]^a.b^.

Both decision-makers and the public at large realize that the gaps between levels of benefits in terms of weekly home care hours and the objective needs of severely dependent elderly should be narrowed. A maximum of twenty two weekly hours seems to be below a reasonable minimum for public LTC program ^a.c^.

Such a position is different from the stand presented by the NII in the earlier days of the program. The first head of the relevant NII department claimed [13]: 103; [14]: 85–86; see also [55] that “the long-term care benefit is not intended to replace the assistance given the severely dependent elderly by his family. Moreover, the modest rate of the benefit testifies to its reliance on the contribution of primary caregivers of the severely dependent elderly in their homes. The services given by family members to the elderly are accepted as self-evident obligations, and there is no payment or other compensation made to the family for these services”. Studies of the respective roles of formal care and non-formal care, mainly family support, of the elderly reached the conclusion that “Generally, formal home care provided by the state was no substitute for family support, but supplemented it” [56]: 25; see also [24,57,58].

Furthermore, even though benefits are targeted at elderly people in need of meaningful help in basic activities of daily living, benefits are not expected to fully cover such individuals’ expenses. A fundamental principle is that “the law should provide a moderate degree of aid to a larger number of elderly persons, rather than providing comprehensive assistance to a more limited number” [13]: 103; [14]: 85; see also [55]. When LTCIP was introduced, two achievements praised by its adherents were that services are allocated based on a legal entitlement, rather than as a voluntary act by the state or non-state actors, and that the number of weekly home care hours increased from 6 on the average ^a.d^ (“which is much less than what is required” [18]: 7) to 10 or 15.

When entitlement rules for LTCIP were set in the 1980s, the NII was well experienced with disability pensions. Thus, disability pensions served as a guide for the new program [10,55]. The (then) two LTCIP benefit levels were defined as percentages of the full disability pension for an individual: 100% and 150%. The number of weekly home care hours does not appear in the law. From the beginning, the number of hours was set by dividing the benefit rates with a fixed tariff for an hour of home care. The tariffs for an hour of home care are updated from time to time by a joint committee of the Ministry of Welfare and Social Services (MOW) and MOF [29]: 636.

Israeli society has changed a great deal since then. The social roles of families as well as expectations from the state in the realm of welfare have changed. The liberalization of Israeli society changed the roles of various sources of welfare such as the family and the state [9,59-62]. Family structures, roles and responsibilities have weakened in the past two decades [58,63,64]. Women, who carry most of the informal care giving responsibilities, are encouraged to join the labor market, thus leaving them with less time and fewer resources to support older family members [65]. While the role of the state as a source of welfare has weakened since the 1980s, socio-economic gaps and poverty in Israel have expanded [1] due to the partial adoption of neo-liberal views and liberalization transferring responsibilities for economic welfare from the state to the market [66,67]. However, public demand for public social services and welfare benefits have not weakened, even if the mix of demands has changed somewhat [68]. On this background, demands for a greater public supply of LTC services for frail elderly have increased over the years [30]: 235–236.

Shifts in political-economic regimes are not linear, coherent, or complete due to program-specific cultural, social and political factors [62]. LTCIP is an example of a welfare state program that seems to change in various directions at the same time – not only in the general direction of a macro-level institutional change of a nation’s political-economic regime. On the one hand, whether current benefit levels meet needs is questionable, and on the other hand, some smaller changes to expand rules of eligibility were introduced in recent years ^a.e^.

Reforming LTCIP benefits faces political obstacles as interests are affected. First, the consent of the MOF is required to promote any reform that increases the total spending on LTCIP. Second, if an increase in spending is not on the agenda, resources should be transferred from the less dependent to the more dependent elderly; that is, reducing the weekly home care hours granted to the less dependent ^a.f^. As the less dependent constitute a large group among the beneficiaries, jeopardizing their vested interests is politically difficult. Third, every change in the benefit scheme affects service providers, which hold political influence over the decision-making process. Fourth, such reforms require amending the NIA, as LTCIP is one section of this Act. The legislation process involves the NII executive board, the government, the Knesset Committee on Labor, Welfare and Health, and the Knesset general assembly.

Since the introduction of LTCIP, benefit levels have been changed several times but none of the changes dramatically increased benefit levels. From April 1988 to August 2000 an additional weekly hour was granted for beneficiaries receiving home care from a not-for-profit service provider. This discrimination was annulled in September 2000. In July 2002 and July 2003, under the Economic Emergency Plan Law and the Israel’s Economic Recovery Law, the MOF promoted cuts in social security programs, including LTCIP ^a.g^.

In January 2007, as the atmosphere of economic crisis that had characterized the beginning of the decade evaporated, a new and slightly more generous benefit was introduced. Changes in LTCIP’s primary and secondary rules may be the response to what is considered as changes in the potential population of beneficiaries (the aging of society and the share of frail elderly), their needs and the required solutions available. The shift from two to three benefit levels in 2007 is an example of a change resulting from the acknowledgment that two levels are not sufficient to meet the growing needs of the frail elderly.

As some changes in LTCIP may increase expenditure, an agreement between the NII and the MOF about many primary legal amendments is required. In 2009 the MOF and the NII reached an agreement on increasing benefits based on a principle other than the needs and incomes of beneficiaries – that is, whether they employ Israeli caregivers – in order to encourage the employment of Israelis rather than foreign workers (see Appendix B). In 2003, when the MOF proposed cutting benefits for elderly people who employ non-Israeli caregivers, the NII opposed the proposal by calling it a “tax” on physically and economically needy persons [69]: 20; see also [70].

Since the introduction of LTCIP, various proposals to restructure benefit levels were put forward and later dismissed, as the NII and MOF could not reach an agreement. At the heart of these proposals was increasing the number of benefit levels, reducing weekly hours for the less dependent and increasing weekly hours for the more dependent [31]. While the NII considered the need to redistribute resources according to need, the MOF opposed proposals that meant increased spending [70,71]. When the NII proposed changes within budgetary limits, the MOF rejected NII’s position that current beneficiaries should enjoy the reforms and demanded to restrict changes to new beneficiaries [72,73], except where cuts were on the agenda [69,70]. It is possible that the MOF was afraid that increasing benefits for the severely dependent beyond a certain threshold would create an incentive for people to demand from the government greater level of responsibility than they demand from other sources of care, such as the family. In 2011, proposals for reform in the benefit level structure of LTCIP were presented by both the NII (with the MOF) and the Ministry of Health (see Appendix A).

Since the introduction of LTCIP, its generosity has not dramatically changed. The number of frail elderly has increased, and among them the share of the severely dependent has rapidly increased – in 2000 22,789 elderly people received the higher of two benefits, while in 2011 the comparable number of those receiving the two higher benefits reached 65,636; from 23.8% of beneficiaries to 45.1% in twelve years [1]: 123. However, benefit levels, as well as the range of services, remained quite intact. Most people eligible for one of the two higher benefit levels require assistance and/or supervision for many hours each day. Some require services for most of the day, or even 24 hours each day. Two more hours per week for less than half of those individuals were added as considerations of greater needs and budgetary constraints met.

A concern of the government linked to increasing LTCIP benefit levels is that such a move would encourage the employment of non-Israeli workers. Formally, the higher benefits grant 16 or 18 weekly hours of home care by non-Israeli caregivers. Yet in fact they cover – whether in-kind or in cash – a meaningful portion of the cost of employment of non-Israeli caregivers, up to a third of the cost ^a.h^. Increasing the highest benefit to 30 weekly hours would cover 60% of the cost of employing a non-Israeli caregiver, and a benefit of 50 weekly hours would cover the entire cost.

Increasing the number of weekly home care hours is required to support the needs of those employing Israeli caregivers, but as long as the legal framework for employing non-Israeli caregivers is not changed, a radical reform in LTCIP benefits would face difficulties. On the one hand, changing the legal framework and equalizing the costs of employing Israelis and non-Israelis would increase the costs of employing non-Israeli workers and increase the financial burden on frail elderly and their families. On the other hand, widening the existing differentiation of benefits based on the citizenship status of the formal caregiver is considered by some as a discrimination against those in need for 12–24 hours a day of formal care, mostly delivered by non-Israelis, even though the real needs covered are much greater in the case of non-Israeli caregivers, as explained above. Others are concerned about the expected costs of increasing benefits to Israeli caregivers. In 2011 the cost of increasing benefits for employing Israeli caregivers was around NIS 195 million, and in 2011 – around NIS 211 million – 5.2% and 5.5% of total expenditure on benefits, respectively [41].

International comparisons of LTC programs and benefit levels are not an easy task. LTC programs in different countries vary regarding the population covered (elderly people or the entire population), the services and their methods of delivery (for example, whether benefits can be paid for care by a relative), assessment methods of disability and dependency, and methods of payment of benefits (in-kind or cash). A comparison of five countries (Austria, Germany, Israel, Japan and the Netherlands) that was carried out about a decade ago showed that Israeli LTCIP benefits at that time were similar in generosity to the other case studies [34]. Two features of the Israeli system stood out as unique in this comparison: benefits in Israel were the most regressive in terms of coverage of needs (see Table 9) and Israel’s LTCIP had only two levels of benefits. Where the number of benefit levels is small, the distribution of resources according to needs becomes less adequate. In the Israeli case, as discussed above, the results are overly generous benefits for the less dependent and not-at-all generous benefits for the severely dependent ^a.i^.

In contrast to Israel’s relatively low benefit levels for the severely dependent elderly, LTCIP covers a relatively high share of beneficiaries among the elderly population. Table 10 presents the situation in Israel in 2008 compared to other OECD countries according to gender and age group. If one adds institutional care, then the share of the elderly (men aged 67 and over and women aged 62 and over) enjoying benefiting from public LTC services increases from 17.5% to about 19.5% ^a.j^.

**Table 10:** LTC users by age and gender, as a share of respective population group (I, II)

**Country**	**Year**	**Female, 65-79**	**Female, 80+**	**Male, 65-79**	**Male, 80+**	**Public LTC spending, share of GDP (%)**
Poland	2008	0.00	0.02	0.01	0.03	0.4
Korea, South	2008	0.02	0.10	0.01	0.06	0.2
Canada	2007	0.01	0.11	0.01	0.07	1.2
Slovenia	2008	0.02	0.14	0.02	0.07	0.8
Ireland	2008	0.01	0.14	0.01	0.09	N/A
Hungary	2008	0.08	0.17	0.06	0.12	0.3
Sweden	2008	0.02	0.18	0.02	0.11	3.6
Iceland	2008	0.02	0.19	0.02	0.13	1.7
Switzerland	2008	0.02	0.21	0.01	0.11	0.8
Netherlands	2007	0.03	0.23	0.02	0.13	3.5
Germany	2008	0.05	0.33	0.05	0.20	0.9
Finland	2008	0.06	0.34	0.05	0.23	1.8
Luxembourg	2007	0.06	0.35	0.05	0.23	1.4
Australia	2007	0.06	0.36	0.03	0.20	0.8
Czech Republic	2008	0.07	0.40	0.05	0.24	0.2
New Zealand	2008	0.10	0.44	0.05	0.27	1.3
Norway	2008	0.08	0.46	0.06	0.32	2.0
Israel (III)	2008	0.13	0.47	0.06	0.32	0.7
Israel’s ranking	-	1	1	1-4	1-2	13
Israel (IV)	2008	0.10	0.47	0.07	0.32	0.7
Israel’s ranking	-	1-2	1	1	1-2	13

Source: NII – Research and Planning Administration; [35]: 41, 46.

Notes: (I) Data for Israel about users refer to LTCIP beneficiaries, only. Data for Israel on public LTC spending as % of GDP refer to 2010 and include community and institutional care ([2]: 90).

(II) Data for Israel includes beneficiaries of home-based care only. For countries other than Israel, see notes in [35].

(III) For Israel, the population of women and men includes those aged 65 and over who receive LTC services in the community under LTCIP.

(IV) For Israel, the retirement age for women is 62 and the retirement age for men is 67.

The brief comparative discussion demonstrates that LTCIP remains rather resilient as far as the expectations of beneficiaries are concerned; benefits are targeted at wide populations with the aim of covering a portion of their needs. Yet, from a distributive justice point of view, resources are not adequately distributed according to need.

## Conclusions: LTCIP goals and need for reform

The introduction of LTCIP generated several institutional changes in the realm of LTC in Israel. Home-based services in the community were limited in the mid-1980s. In 1986 about 5,000 elderly people received some home care services from the (then) Ministry of Labor and Welfare, the Ministry of Health and Kupat Holim Clalit [13]: 102; [18]: 7. In 1989 over 21,000 elderly people received more services under LTCIP. Thereafter, growth rates in the number of beneficiaries outpaced the growth rates in the number of elderly people in Israel. The introduction of LTCIP has generated the evolution of a multi-player not-for-profit and for-profit, service providers. The state has financed the development of a robust and competitive service system from which frail elderly and their families benefit.

Over the years since the introduction of LTCIP, while the growth in the number of beneficiaries outpaced the growth of the elderly population in Israel, the character of the population of beneficiaries changed reflecting changes in the elderly population in the country as a whole. The relative share of men and women among the beneficiaries has slightly changed and the share of the aged who are more dependent has increased, reflecting the aging of the Israeli elderly in general. LTCIP’s legal rules were amended numerous times, especially in the last decade, and the administrative orders that guide its day-to-day implementation are constantly under review and adapted to meet requests from the public. Even though legal changes and administrative procedures may be meaningful in individual cases for beneficiaries, formal caregivers or service providers, most changes in LTCIP have been rather gradual, and not radical [7]^a.k^. These two aspects – of the law and of the beneficiaries – demonstrate the basic resilience and stability of this welfare state program.

The introduction of LTCIP has shifted the burden of LTC services from focusing on institutional care to the community and home care. LTCIP has not narrowed the use of institutional solutions, but in recent years the growth rate in the number of beds in LTC institutions has become more moderate. From 1990 to 2000 the number of beds increased by 47.7%, from 19,041 to 28,131. At the same time, the growth rate of the aged 75+ outpaced the growth rate of the number of beds, as the ratio of beds per 1,000 aged 75+ fell from 103 to 102. The decrease in this ratio was even sharper in the 1980s – from 113 to 103. From 2000 to 2009, the number of beds increased by only 4.1% ^a.l^. The beds per 1,000 aged 75+ ratio fell to 83 [3]: 326. It seems that the growth in rate of elderly people enjoying LTCIP has contributed to this trend.

One of the contributions of LTCIP – even if it was not a goal of the program originally – was the employment of many individuals who otherwise would have faced difficulties in the labor market [14]: 95–96. Most of these caregivers are non-professional, part-time and temporarily hired, and low-paid workers. Most of them are women and many immigrated to Israel since 1990. The status and income of these formal caregivers is an issue that has been considered by decision-makers [30,31,74].

One cannot ignore the contribution of LTCIP to meeting the needs of frail elderly people in Israel. In that respect, LTCIP contributed to the solidarity between the aged and the rest of society as it met some of the needs of many frail elderly [13]: 112. Yet, as Israeli society, and its fundamental institutions such as the family, changes over time, the question of whether LTCIP’s goals, rules of eligibility, and methods and levels of benefits are adequate or need to be adapted is under continuous public debate.

The article raises several issues that decision-makers should pay attention to, and many of these issues are currently on the policy agenda ^a.m^. First, it seems that some reforms in the rules of LTCIP, especially the structure of benefit level, are required. Other than the political difficulties linked to the implementation, there is uncertainty about the implications for the behavior of clients and of service providers. Second, the growing number of frail elderly people and the expected aging of the Israeli society have implications for the financial stability of LTCIP and that of the NII in general. Perhaps the methods of financing of LTCIP, such as raising NII insurance fees dedicated to LTCIP, should be evaluated. Reforming the structure of the benefit levels may have future financial implications, since the behavior of clients and service providers and their interactions with the NII may change once the rules are changed.

Other issues are linked to the brief discussion in Appendix B. First, the NII should develop mechanisms to evaluate and supervise the quality of care under LTCIP and enhance its regulatory role, rather than leaving the supervision to the claimants/beneficiaries and their families or to the service providers themselves. Second, Appendix B points to the place of foreign caregivers and the aging of Israeli caregivers. A wide use of foreign caregivers has social implications that might contradict targets related to the scope and quality of care and restraining expenditures. To sum up, the decision on whether or not to adapt LTCIP to the changing needs of the elderly has implications not only on the elderly in Israel, but on Israeli society as a whole.

Finally, the analysis raises several topics for future research, such as the changes in the respective roles of formal care and family support for the elderly within a liberalized welfare state, the factors (socio-demographic and economic as well as policy implementation) that affect access to LTCIP and take-up rates and the influence of globalization of the labor market on the relationships of beneficiaries and families, service providers and formal caregivers. This paper did not address some of the political aspects of LTCIP that deserve further research, such as the satisfaction of claimants and beneficiaries from LTCIP and public support of LTCIP and NII and their effects on the mechanisms through which the NII, service providers, and the public interact to change LTCIP and the distribution of resources to LTCIP and within LTCIP.

## Endnotes

^a^ Some of these studies focus on the implications of LTCIP on beneficiaries and their family members (informal caregivers) [22,24,56,57,75-78], formal caregivers [27,78] and service providers [26,79-85]. Several studies and government reports have considered the place of foreign caregivers [86-93].

^b^ A unique place is given to the issue of method of payment of benefits – whether in-kind or in cash [13-16,25,31-33,65]; see also [19].

^c^ Other important measurements of success such as satisfaction of beneficiaries, claimants and family members from LTCIP and the program’s public support deserve separate studies.

^d^ In the Israeli case, private LTC insurance schemes are offered by the four Sick Funds (not-for-profit medical services providers and insurers responsible for delivering services listed under the National Health Insurance Act, 1994) via insurance firms and by 8 insurance firms (as of 2010). Private LTC insurance is not conditioned by or linked to eligibility to LTCIP, and a person can receive benefits from public and private sources at the same time. These insurance schemes cover both the elderly and younger people. The private insurance schemes differ by their premium levels (which are age-related), rules of eligibility (including LTC needs, based on an ADL evaluation, and previous medical conditions to some extent), waiting times, periods of benefits, and levels of benefits for home care and institutional care [94]; see also [95]. The insurance firms offering these plans claim that they are more generous than the public LTCIP, thus better meeting the growing needs of frail elderly people. While levels of benefits for home care, whether in cash or in-kind, are in most cases higher in these programs than in the LTCIP, the premiums are usually higher than the national insurance fees for LTCIP.

^e^ The cost of long-term institutionalization is shared by the Ministry of Health (MOH) and the Ministry of Welfare and Social Services (MOW) and copayments [2,17].

^f^ The government finances the cost of benefits for Jewish immigrants who are not insured under the law; in recent years the share of this group out of total benefit recipients has slightly decreased – to about a fifth. Since 1990, the government also pays the NII a share of the employers’ contributions to LTCIP as part of its policy to ease the tax burden on the employers in order to reduce labor costs.

^g^ The rules of income/means-testing used by the MOW and the MOH for public financing of institutional care are much stricter, and include income and means testing of the elderly and their spouses and income testing of children and children’s spouses.

^h^ Whether or not LTCIP can still be considered a social security program cannot be answered easily. On the one hand, legally, it is defined as a social security program and one of its sources of funding is insurance fees paid by employees and employers. On the other hand, the share of insurance fees in its financing has decreased over time, as presented below. Moreover, while legal amendments to LTCIP with budgetary implications require the agreement of the Ministry of Finance (see below), the public – claimants, beneficiaries and their relatives were successful in pushing the National Insurance Institute to introduce changes to other parts of the program such as the dependency evaluation process – and the politicians to introduce several legal changes regarding rules of eligibility (see below).

^i^ It was introduced due to budget constraining considerations [96] at about the same time that income-testing was included in the child allowance scheme. Income-testing in the child allowance scheme was abolished in 1993.

^j^ I wish to thank Brenda Mroginstin, who was involved in formulating the rules of LTCIP, for pointing out to me some of the considerations that shaped the principles of dependency test when it was first included in LTCIP. The decisions to include ADL but not IADL, and to limit the definitions of dependency to a need of help of another person, but not the use of durable medical equipment, resulted from a need to harmonize between contradicting professional considerations and economic constrains – to identify the needy and to restrain expected costs.

^k^ In most cases the use of durable medical equipment, such as cane or a walker, do not grant a score in the dependency test, but the use of a wheelchair does.

^l^ The ADL section in the dependency test used in LTCIP was developed in the Attendance Allowances Program in the NII that grants cash benefits to disabled individuals who require assistance in ADL [97]: 34. Over the years, the dependency tests in the two programs diverted from one another in their internal rules. The ADL section in the dependency test in LTCIP has some resemblance to the Katz ADL index [98], although they are not identical in the definition of activities considered or the scoring method (see Table 1) [99]. The activities “going to toilet” and “continence” are combined under “control of urine and bowel movements”. The activity “transfer” is one part of mobility in the home. Also, the dependency test includes a section aimed at evaluating the need for supervision [43].

^m^ The dependency score for eating and cooking includes assistance in taking medications.

^n^ Since its introduction, the dependency test in LTCIP included ADL and the need for supervision. Inclusion of IADL (instrumental activities of daily living) or expanding the criteria of ADL to include mobility outside the home are expected to increase the number of beneficiaries and total expenditure [3]: 124–125; [10]; see also [100]

^o^ The section in the dependency test that examine the need of supervision was thoroughly reviewed in the late 1990s and early 2000s following recommendations of committees staffed by experts in psycho-geriatric medicine and NII officials [46,101].

^p^ The use of “radical” or “moderate” to evaluate changes in a social program such as LTCIP draws from J.L. Campbell’s conceptualization of “institutional change”, which is based on the researcher’s identification of the central dimensions of an institution and a decision of the right time frame for evaluation of change’ or lack of it, i.e. stability [7]. In the case of LTCIP, the evaluation of the magnitude of change is based on identification of the principles of the program and how amendments changed them or affected beneficiaries since their introduction.

^q^ Codex, No. 2077, 11.1.2007, p. 52.

^r^ Codex, No. 2080, 1.2.2007, pp. 108–109.

^s^ Codex, No. 2203, 23.7.2009, pp. 248–249.

^t^ Attendance allowances are cash benefits. In December 2011, 7,179 elderly people received attendance allowances compared to 6,574 in December 2010 [53].

^u^ Bills, No. 169, 23.7.2007, p. 270; Bills, No. 292, 14.12.2009, p. 60; Bills, No. 301, 19.1.2010, p. 89; Bills, No. 405, 25.7.2011, p. 236; Bills, No. 619, 19.9.2011, p. 1628; Codex, No. 2139, 18.3.2008, pp. 252–253; Codex, No. 2225, 4.2.2010, p. 327; Codex, No. 2277, 17.2.2011, p. 355; Codex, No. 2310, 11.8.2011, p. 1024; Codex, No. 2331, 12.1.2012, pp. 110–111.

^v^ One can compare the development of LTCIP to other benchmarks: during 1989–2009 the number of elderly people aged 65 and over increased 1.8 times [52]; during 1991–2010 the entire population of Israel increased by 1.5 times and Israel’s national expenditures for health-care increased 2.1 times [102]: 50.

^w^ MOF contributions finance LTCIP benefits for uninsured immigrant elderly.

^x^ As mentioned above, during 2004–2008 the retirement ages for women and men were gradually raised from 60 to 62 and from 65 to 67, respectively.

^y^ Another explanation, not studied in this paper, might be the role of institutions for elderly women and men, and which the men and women choose, or their families choose for them.

^z^ Since part of the gap is due to a variation in the minimum age for eligibility between women and men – among beneficiaries who became eligible for the first time at the age of 67 or over, men first become eligible at the age of 78.8 while women first become eligible at the age of 77.1.

^a.a^ The average duration of eligibility for LTCIP is calculated based on the records of beneficiaries who entered the LTCIP system starting January 1998, or later, and exited the system due to death, moving to an institution, or their eligibility was temporary (a total of 96,837 records).

^a.b^ I wish to thank Orna Zamir and Roni Dinur from the LTC Department of the NII for pointing this out to me.

^a.c^ Holocaust survivors who receive one of the two highest LTCIP benefits also receive additional home-based care in the amount equivalent to 9 weekly hours from The Foundation for the Benefit of Holocaust Victims in Israel [103]. This is a voluntary non-state arrangement.

^a.d^ Services provided by the Ministry of Labour and Social Affairs, the Ministry of Health and the General Sick Fund in the pre-LTCIP era [18].

^a.e^ See the discussion in the section “LTCIP: an overview” in this paper.

^a.f^ In recent years, the position of the LTC Department of the NII has been that beneficiaries with 2.5-3 dependency points enjoy greater number of weekly home care in relative to their needs. This impression is based on comments from beneficiaries themselves as well as from their relatives who cannot keep the formal caregivers occupied for the entire time. I wish to thank Orna Zamir and Roni Dinur from the LTC Department of the NII for pointing this out to me.

^a.g^ In July 2002 the lower benefit level was cut from 11 to 10.5 weekly hours and the higher level was cut from 16 to 15.5 weekly hours. In July 2003 the lower level was cut from 10.5 to 9.75 weekly hours.

^a.h^ The monthly cost of employing a non-Israeli caregiver was 7,500 NIS in 2010 [92]. The legal framework covering the employment of non-Israeli caregivers maintains that they are not eligible for overtime pay [104]. In 2010, the value of the highest in-kind monthly benefit was NIS 3,392, and that of cash benefit was NIS 2,714. This means that the LTCIP benefit covers up to a third of the monthly cost of employing a non-Israeli caregiver (see also [20]: 687). Thus, there are incentives for hiring foreign caregivers.

^a.i^ Based on a comparison between Israel (see Table 9) and Germany. For Germany see [105]: 72; [106]: 46–47.

^a.j^ In 2008 there were about 15,400 public subsidized beds in institutions for LTC for the aged [107,108]. With addition of elderly recipients of attendance allowances (5,793 in December 2008; see [109]: 220), the share of elderly (men aged 67 and over and women aged 62 and over) enjoying public LTC services and/or funding is 20.3%.

^a.k^ For example, the introduction of cash benefits has been limited to only 9 of the 23 local NII branches under an “experimental program” and only 8% of the potential beneficiaries in the four branches where the program started in March 2008 opted to receive cash benefits by the end of 2011 [1]: 132–134.

^a.l^ The number of beds reached a peak in 2004 – 30,775 [110]: 298.

^a.m^ For reforms in the structure of benefit level, see above. For efforts to evaluate the financial implications of LTCIP on the NII in the future, see [111]. Regarding regulating quality of care, this issue is at initial stages of development at the NII. For some of the measures used to restrain the employment of foreign workers and encourage Israeli workers in LTC, see Appendix B.

^a.n^ In [112] an earlier version of the reform proposal was published in the press.

^a.o^ Figures were adapted to available data for March 2012. Source: NII – Research and Planning Administration.

^a.p^ According to the director general of MATAV, the largest not-for-profit service provider of LTC services, the reform proposal may grant the sick funds too much power and might lead to a conflict of interests between the sick funds’ responsibilities of management of care and provision of services against the interests of the beneficiaries. A senior management at DANEL, one of the largest for-profit service providers of LTC services, was concerned that transferring responsibilities to the sick funds would not benefit the beneficiaries. See summary of these comments in [113].

^a.q^ Such concerns were raised by the Director General of the NII, the Deputy Head of the Budget Department at the MOF and senior managers of Maccabi Sick Fund (the second largest sick fund) at the 14^th^ Seminar in the Memory of Rafi Rotter held at November 22, 2012 and focused on the reform proposal of the MOH.

^a.r^ In the proposal, the addition to the final dependency score for people living alone is different than the current situation, thus comparing the change in benefits for individual cases is not intuitive. Also, the maximum dependency score is 10.5, not 11, according to this proposal [1].

^a.s^ See data regarding Israeli formal caregivers below.

^a.t^ Schmid and Borowski found that in the late 1990s the formal caregivers’ average age was 43 [27]: 94.

## Appendix A

Ministry of Health (MOH) and NII-MOF joint LTCIP reform proposals

The NII and the MOF held discussions following an initiative of the NII to restructure benefits according to perceived needs by increasing benefits for the severely dependent at the expense of the less dependent ^a.n^. Table 11 presents this reform proposal. Even though the joint NII-MOF proposal does not eliminate the nonlinearity problem, it still increases benefits as levels of dependency rise, and it offers more assistance for those in greater need.

**Table 11:** Reform proposal discussed by the NII and the MOF during 2011)

**Dependency score **^**a.r**^	**Non-Israeli caregiver**	**Israeli caregiver**
2.5	6	6
3	6	6
3.5	9	9
4	9	9
4.5	9	9
5	12	15
5.5	12	15
6	12	15
6.5	16	20
7	16	20
7.5	16	20
8	21	25
8.5	21	25
9	21	25
9.5	26	29
10	26	29
10.5	26	29

Source: [1]: 13

This proposal was vaguely incorporated into the Trachtenberg Report [114]: 143–144 following the “social unrest” of summer 2011 in Israel. While benefits for the less dependent are reduced by up to 3.75 weekly hours, benefits for the more dependent are increased by up to 8 weekly hours. The main criticism of the proposal was raised by the MOH, as 55.1% of beneficiaries are expected to experience a decrease in care services while only 38.1% will enjoy an increase [115]^a.o^. The reason is that more beneficiaries are less dependent.

Recently, the MOH proposed to reform the entire LTC system by consolidating services under the responsibility of the sick funds and to increase the number of weekly home care hours for frail elderly based on their scope of dependency and incomes [2]; see also [17]. According to this proposal, benefits would be increased to up to 33 weekly home care hours, higher benefits (additional 2.5-10 weekly hours) would be granted for employing Israeli caregivers, benefits for the less dependent would not be reduced, income-testing would be terraced – but the program would cover the highest income earners as well – and additional spending would be allocated. Such a reform is expected to increase total expenditure for all LTC services – in the community and institutional – and to mobilize opposition from major stakeholders such as the NII, service providers ^a.p^, and the sick funds expected to be affected by it [113], as well as the MOF. The NII is concerned that the proposal of the MOH may jeopardize the quality of care provided for the elderly, while the MOF is worried that the financial stability of the sick funds might be at risk. The sick funds have been reluctant to support this initiative due to a possible scenario of insufficient funding ^a.q^.

## Appendix B

The “LTC industry” – service providers and formal caregivers

The introduction of LTCIP generated the development of what several commentators called the “LTC industry” [25]: 197; [79]: 182. The LTC industry includes various for-profit and not-for-profit service providers delivering LTCIP services. Prior to LTCIP, only one, not-for-profit, organization – MATAV – handed LTC services for the elderly. Following LTCIP, the number of service providers has increased dramatically. These organizations employ a growing number of Israeli caregivers or foreign LTC laborers. In August 2011, 112 LTC companies delivered home care services under LTCIP. Forty-six companies were not-for-profit, but the share of home care hours delivered by the other 66 for-profit companies reached 72.2% in August 2011. The share of home care hours delivered by for-profit companies is on the rise: their share of total home care hours, which started at 49% in 1989, increased to 60.1% in 1995, to 69.5% in 2002, and to 70.8% in 2009 [53].

LTCIP is arranged in a format of quasi-markets where the state sets standards and prices while service providers compete over beneficiaries and quality of services within fixed prices [16]: 55. The supervising role of the state over the quality of services delivered is a modest one. The increase in the number of beneficiaries and of service providers over the years has limited the capabilities of the NII and the local LTC committees to supervise their operations and quality of care [15]: 37; [28]: 606; [30]: 245–249. LTCIP is based on the assumption that multiple service providers and competition among them is the best way to ensure adequate services, as beneficiaries can ask the service provider to change their formal caregiver or to switch to another service provider altogether.

A common perception about LTC for the elderly in Israel claims that most formal caregivers are foreign workers. According to estimations published by the OECD, the share of foreign workers in Israel’s LTC sector’s workforce reached 50% in 2010 [35]: 174. In December 2011, 36,600 LTC beneficiaries held a valid permit for employing a foreign worker compared to 36,700 beneficiaries in December 2010. During 2011, the total share of holders of a valid permit among all beneficiaries fell from 25.5% to 24.5%. Similar drops can be noticed in all benefit levels (see Table 4). About one-third of formal caregivers are foreign workers ^a.s^.

Since March 2009 the weekly home care hours for those eligible for LTCIP benefits at one of the two higher levels was increased by 3 and 4 hours, respectively. These additions might postpone the need to hire a foreign caregiver, available for up to 24 hours a day, for those beneficiaries who need care for only several hours every day. However, it seems that the main cause of the freeze in the number of foreign workers in the LTC sector has been the government’s policy since June 2010 of setting quotas for the number of LTC foreign caregivers that agencies may bring to Israel, based on the rates of their success in assigning LTC foreign workers already in Israel [116,117].

The number of Israeli formal caregivers increased from about 20,000 in the early 1990s [14], to 50,000 at the early 2000s [15] to almost 70,000 in December 2011. The majority of formal caregivers – 92.8% – are women, and many of them – 43.9% – immigrated to Israel since 1990. As can be figured by their occupation, most are non-professional, low-paid, part-time, and temporary employees who belong to the lower strata of Israeli society.

The mean age of Israeli formal caregivers is 48.2, and their average age has risen over the years ^a.t^. Female caregivers’ average age is lower than that of male caregivers – 48 compared to 50.4. Post-1990 immigrant caregivers’ average age is much higher than the average age of the other caregivers – 52.8 compared to 44.5. The oldest group among Israeli caregivers is that of male post-1990 immigrants – 56.8. The average age of female post-1990 immigrant caregivers is 52.5. The average age of female and male caregivers born in Israel or immigrated before 1990 is similar – 44.6 and 44.3, respectively.

## Abbreviations

CBS: Israel Central Bureau of Statistics; LTC: Long-Term Care; LTCIP: Long-Term Care Insurance Program; MOF: Israel Ministry of Finance; MOH: Israel Ministry of Health; MOI: Israel Ministry of The Interior; MOW: Israel Ministry of Welfare and Social Services; NIA: National Insurance Act; NII: Israel National Insurance Institute; NIS: New Israeli shekel (Israel’s currency).

## Competing interests

The author declares that he has no competing interests.

## Author’s information

Sharon Asiskovitch is a social policy analyst at Research and Planning Administration at the National Insurance Institute of Israel focusing on Long-Term Care Insurance Program. He received his doctorate in Political Science from the Hebrew University of Jerusalem in 2007.
